# Neutrophil to lymphocyte ratio and breast cancer risk: analysis by subtype and potential interactions

**DOI:** 10.1038/s41598-020-70077-z

**Published:** 2020-08-06

**Authors:** Manuela Gago-Dominguez, Marcos Matabuena, Carmen M. Redondo, Sandip Pravin Patel, Angel Carracedo, Sara Miranda Ponte, María Elena Martínez, J. Esteban Castelao

**Affiliations:** 1 Galician Public Foundation of Genomic Medicine (FPGMX), Genomic Medicine Group, International Cancer Genetics and Epidemiology Group, Health Research Institute of Santiago (IDIS), Santiago de Compostela, Spain; 2 Moores Cancer Center, University of California San Diego, La Jolla, CA USA; 3 Centro de Investigación en Tecnoloxías da Información (CITIUS), Universidade de Santiago de Compostela, Santiago de Compostela, Spain; 4 Oncology and Genetics Unit, Instituto de Investigación Sanitaria Galicia Sur, Vigo, Spain; 5 Forensic Department, Universidade de Santiago de Compostela, Santiago de Compostela, Spain; 6 Department of Family Medicine and Public Health, University of California San Diego, La Jolla, CA USA

**Keywords:** Lipidomics, Breast cancer, Biomarkers, Breast cancer

## Abstract

Multiple studies have found the neutrophil to lymphocyte ratio (NLR) to be associated with adverse breast cancer (BC) prognosis and survival. Very limited data exist on the role of NLR and risk of BC. The BREOGAN study is a population-based case–control study conducted in Galicia, Spain. We examined the WBC- and NLR-BC relationships. The risk of BC increased with increasing levels of neutrophils percentage (NE%) (multivariable OR for the highest category (95% CI) = 2.14 (1.39–3.32), P-trend < 0.001) and of the NLR (multivariable OR for the highest category (95% CI) = 1.93 (1.26–2.97), P-trend < 0.001). Lymphocytes absolute (L#) and percentage (L%) were associated with a decreased risk of BC (multivariable OR for the highest category (95% CI) = 0.54 (0.35–0.83), and 0.51 (0.33–0.79), P-trend = 0.001 and < 0.001, respectively). The NLR-BC association was more pronounced among Luminal A BC (multivariable OR for the highest category (95% CI) = 2.00 (1.17–3.45), P-trend < 0.001), HER2-negative BC (multivariable OR for the highest category (95% CI) = 1.87 (1.16–3.02), P-trend < 0.001), and those with high total cholesterol and low H_2_O_2_ levels.

## Introduction

In 2018, 32,825 new breast cancer cases were diagnosed in Spain, representing 12% of all cancer cases, and 29% of all cancers in women^1^. It was responsible for 6,421 deaths (6% of all cancer deaths). Breast cancer is the most frequent cancer in Spain and ranks the fourth in cancer-related mortality. There has been a 30% increase in breast cancer incidence between 2012 and 2018^1^.

High concentration of blood neutrophils is seen in patients with advanced cancer and are associated with poor survival^2,3^. Similarly, there is abundant evidence for an adverse prognostic value of neutrophil to lymphocyte ratio (NLR) on breast cancer. Multiple studies have shown that higher NLR was associated with poorer survival^4–9^, and a recent meta-analysis found that higher NLR was associated with both worse disease-free survival and overall survival^6^. Several previous studies have found that higher NLR was also associated with more advanced or aggressive breast cancer^3,7,10,11^.

For this reason, the ratios between neutrophils in blood and other leukocytes, as the NLR, have been suggested as a prognostic value in cancer^3,12,13^. NLR is higher in patients with more advanced cancers^10^, and correlates with poor survival in many cancers^14,15^. Thus, NLR, a simple and inexpensive biomarker, has been introduced as a significant prognostic factor in many tumour types^3^. However, it has not been accepted in many clinical settings since neutrophilia can be also the result of elevated granulopoiesis and, therefore, may not be an adverse sign for cancer progression^3^. Another reason is that neutrophilia is associated with poor clinical outcome in all cancers except one, stomach cancer, in which a high NLR is a marker of good prognosis^3,16^. However, the evidence for a detrimental effect of circulating neutrophils and the NLR on cancer prognosis and survival altogether, with only the exception of gastric cancer, is very consistent.

To our knowledge, only three small studies, conducted in Asian women, assessed the association between NLR and the risk of breast cancer^7,17,18^. Two of the three studies compared breast cancer cases with benign breast disease (BBD) controls^7,17^, and the third one used healthy controls as reference group^18^. In one of the studies^17^, the NLR was significantly higher in breast cancer patients compared with the BBD group, and patients with NLR > 1.67 were related to an increased risk of breast cancer. This finding was consistent with the other two studies^7,18^, suggesting that NLR could be an independent risk factor for breast cancer.

In the present study, we examined the association of WBC including neutrophils, lymphocytes and monocytes, and the NLR, on the risk of breast cancer overall, and by major subtypes, menopausal status, and grade and morphology, in Spanish women.

## Results

Table 1 shows the individual associations between established risk/protective factors for breast cancer and risk of breast cancer in the present study. Similar to previous studies^19–21^, obesity was associated with an increased risk of breast cancer (multivariable OR (95% CI) = 2.02 (1.30–3.17), P-trend = 0.003). Use of oral contraceptives was also associated with an increased risk of breast cancer (multivariable OR (95% CI) = 2.01 (1.22–3.30)). Similarly, ex-smokers and current smokers were associated with an increased risk of breast cancer (multivariable OR (95% CI) = 2.18 (1.14 – 4.14), and 1.86 (0.87–3.93), for ex-smokers and current smokers, respectively). A history of first- and/or second- degree relatives with breast and/or ovarian cancer was associated with an increased risk of breast cancer (multivariable OR (95% CI) = 2.46 (1.49–4.06)). Consistent with previous studies, invasive ductal carcinoma was the most frequent histological type (80%), and the distribution of the Luminal A, Luminal B, HER2 overexpressed, and TNBC subtypes were 63%, 13%, 8% and 15%, respectively. Mean tumor size in cm was 2.1.​

**Table 1:** Associations between risk/protective factors for breast cancer and breast cancer risk.

	Cases N (%)	Controls N (%)	OR^a^	95% CI	OR^b^	95% CI
N	300	372				
Mean age, year	55.5 ± 13.1	61.0 ± 13.5				
**Age at menarche, year**						
≤ 12	91	101	1.00		1.00	
> 12	209	271	0.95	0.67–1.34	1.05	0.73–1.51
**Age at menopause, year**						
> 50	85	123	1.00		1.00	
≤ 50	81	142	0.84	0.56–1.25	0.91	0.60–1.37
**Family history**^**c**^						
No	49	181	1.00		1.00	
Yes	89	82	2.52	1.56–4.09	2.46	1.49–4.06
**Age at first full time pregnancy, year**						
≤ 25	49	170	1.00		1.00	
26–30	25	72	0.96	0.52–1.73	0.99	0.54–1.80
> 30	29	32	1.60	0.81–3.15	1.67	0.84–3.29
P-trend			0.75		0.66	
**Number of pregnancies**						
0	49	46	1.00		1.00	
1–2	195	185	1.32	0.82–2.14	1.25	0.76–2.07
≥ 3	55	119	0.73	0.41–1.29	0.71	0.39–1.28
P-trend			0.10		0.054	
**Lifetime breastfeeding, months**^**d**^						
0	13	73	1.00		1.00	
< 12	52	88	2.59	1.27–5.56	2.70	1.30–5.91
≥ 12	10	59	0.98	0.37–2.54	1.01	0.37–2.66
P-trend			0.42		0.45	
**Body mass index, kg/m**^**2**^						
< 25	100	125	1.00		1.00	
25–29	102	142	1.21	0.82–1.79	1.23	0.82–1.84
≥ 30	88	83	1.92	1.25–2.97	2.02	1.30–3.17
P-trend			0.004		0.003	
**Oral contraceptive use**						
Never	55	207	1.00		1.00	
Ever	82	96	1.77	1.10–2.85	2.01	1.22–3.30
**Smoking status**						
No	60	214	1.00		1.00	
Ex-smoker	26	29	2.13	1.12–4.03	2.18	1.14–4.14
Current smoker	20	20	1.85	0.87–3.89	1.86	0.87–3.93
**Hormone replacement therapy**						
Never	118	262	1.00		1.00	
Ever	15	44	1.06	0.54–2.00	1.08	0.54–2.08
**Histology type**						
Ductal	232 (80.3)					
Lobular	27 (9.3)					
Mucinous	8 (2.8)					
Mixed	9 (3.1)					
Other	13 (4.5)					
**Tumor size, cm**	2.1 ± 1.6					
**Breast cancer subtypes**						
Luminal A	168 (63.2)					
Luminal B	36 (13.5)					
TNBC	40 (15.0)					
HER2 overexpressed	22 (8.3)					

^a^Adjusted for age at diagnosis (cases) and age at interview (controls).

^b^Further adjusted for age at menarche, parity, menopausal status, and BMI.

^c^Defined as one or more first and/or second-degree relatives with breast and/or ovarian cancer.

^d^Among parous women only.

Table 2 presents the risk of breast cancer according to WBC and NLR levels. The risk of breast cancer significantly increased with increasing circulating levels of neutrophils absolute (NE#) and neutrophils percentage (NE%) (multivariable OR for the highest versus the lowest category (95% CI) = 1.40 (0.92–2.15) and 2.14 (1.39–3.32), P-trend = 0.02 and < 0.001, respectively). Similarly, lymphocytes absolute (L#) and lymphocytes percentage (L%) were associated with a decreased risk of breast cancer (multivariable OR for the highest versus the lowest category (95% CI) = 0.54 (0.35–0.83), and 0.51 (0.33–0.79), P-trend = 0.001 and < 0.001, respectively). No other WBC component was significantly associated with risk of breast cancer. The risk of breast cancer significantly increased with increasing levels of the neutrophil to lymphocyte ratio (NLR), calculated by dividing the number of neutrophils by number of lymphocytes (multivariable OR for the highest versus the lowest category (95% CI) = 1.93 (1.26–2.97), P-trend < 0.001). The risk of breast cancer significantly decreased with increasing levels of hydrogen peroxide (H_2_O_2_) (multivariable OR for the highest versus the lowest category (95% CI) = 0.26 (0.13–0.51), P-trend < 0.001).

**Table 2:** Circulating white blood cells, neutrophil to lymphocyte ratio (NLR), H_2_O_2_ and risk of breast cancer.

	Cases/Controls	OR^a^	95% CI	OR^b^	95% CI
**Neutrophils #, 10**^**3**^**/μL**					
< 2.8	72/120	1.0		1.0	
2.8–3.7	100/123	1.26	0.85–1.89	1.17	0.77–1.79
> 3.70	127/113	1.66	1.12–2.47	1.40	0.92–2.15
P-trend			0.002		0.02
**Neutrophils %**					
< 48.6	54/121	1.0		1.0	
48.6–57	109/127	1.88	1.24–2.86	1.79	1.17–2.78
> 57	136/111	2.42	1.60–3.68	2.14	1.39–3.32
P-trend			< 0.001		< 0.001
**Monocytes #, 10**^**3**^**/μL**					
< 0.3	82/131	1.0		1.0	
0.3–0.44	114/108	1.59	1.08–2.34	1.61	1.06–2.43
> 0.44	103/117	1.43	0.97–2.11	1.36	0.90–2.07
P-trend			0.18		0.38
**Monocytes %**					
< 5.87	118/125	1.0		1.0	
5.87–7	108/150	0.86	0.60–1.23	0.95	0.65–1.40
> 7	73/84	1.02	0.68–1.54	1.06	0.68–1.64
P-trend			0.16		0.17
**Lymphocytes #, 10**^**3**^**/μL**					
< 1.9	136/133	1.0		1.0	
1.9–2.5	107/119	0.85	0.59–1.22	0.93	0.63–1.36
> 2.5	56/104	0.52	0.34–0.78	0.54	0.35–0.83
P-trend			< 0.001		0.001
**Lymphocytes %**					
< 31	141/124	1.0		1.0	
31–39	106/123	0.79	0.55–1.13	0.87	0.59–1.27
> 39	52/111	0.44	0.29–0.67	0.51	0.33–0.79
P-trend			< 0.001		< 0.001
**NLR**					
< 1.26	58/117	1.0		1.0	
1.26–1.85	101/121	1.64	1.08–2.49	1.50	0.97–2.33
> 1.85	139/117	2.19	1.46–3.30	1.93	1.26–2.97
P-trend			< 0.001		< 0.001
**H**_**2**_**O**_**2**_** #, ng/mL**					
< 284	78/52	1.0		1.0	
284–517	29/48	0.42	0.23–0.75	0.42	0.22–0.79
> 517	19/49	0.25	0.13–0.47	0.26	0.13–0.51
P-trend			< 0.001		< 0.001

^a^Adjusted for age at diagnosis (cases) and age at interview (controls).

^b^Further adjusted for age at menarche, parity, menopausal status, and BMI.

Table 3 shows the NLR-breast cancer association by the main four breast cancer subtypes and menopausal status. The NLR-breast cancer association was more pronounced among Luminal A breast cancers (multivariable OR for the highest category (95% CI) = 2.00 (1.17–3.45), P-trend < 0.001), although we lacked precision for other breast cancer subtypes. Stratified analysis based uniquely on HER2 receptor status showed that the NLR-breast cancer association was more pronounced among HER2-negative breast cancers (multivariable OR (95% CI) = 1.87 (1.16–3.02), P-trend < 0.001). The NLR-breast cancer association was present in both, pre- and postmenopausal breast cancer, although it was more pronounced among postmenopausal breast cancer (multivariable ORs for highest category of NLR (95% CI) = 1.65 (0.69–3.92), and 2.06 (1.23–3.50), P-trend = 0.19 and < 0.001 for premenopausal and postmenopausal breast cancer, respectively) (Table 3).

**Table 3:** Neutrophil to lymphocyte ratio (NLR) and breast cancer risk by breast cancer subtypes and menopausal status.

	Cases/Controls	OR^a^	95% CI	OR^b^	95% CI
**By breast cancer subtypes:**					
**Luminal A**					
< 1.26	30/117	1.0		1.0	
1.26–1.85	61/121	1.91	1.16–3.20	1.76	1.03–3.05
> 1.85	74/117	2.34	1.43–3.90	2.00	1.17–3.45
P-trend			< 0.001		< 0.001
**Luminal B**					
< 1.26	7/117	1.0		1.0	
1.26–1.85	11/121	1.32	0.48–3.78	1.10	0.39–3.24
> 1.85	18/117	2.02	0.81–5.50	1.83	0.70–5.15
P-trend			0.049		0.08
**TNBC**					
< 1.26	12/117	1.0		1.0	
1.26–1.85	8/121	0.62	0.23–1.57	0.59	0.22–1.54
> 1.85	19/117	1.46	0.68–3.25	1.56	0.70–3.59
P-trend			0.03		0.03
**HER2-overexpressed**					
< 1.26	3/117	1.0		1.0	
1.26–1.85	13/121	3.91	1.21–17.48	3.63	1.10–16.45
> 1.85	6/117	1.76	0.45–8.57	1.80	0.45–8.90
P-trend			0.77		0.79
**By HER2 status:**					
**HER2-negative**					
< 1.26	42/117	1.0		1.0	
1.26–1.85	70/121	1.58	0.99–2.51	1.42	0.88–2.33
> 1.85	96/117	2.15	1.38–3.39	1.87	1.16–3.02
P-trend			< 0.001		< 0.001
**HER2-positive**					
< 1.26	10/117	1.0		1.0	
1.26–1.85	25/121	2.22	1.02–5.14	2.02	0.92–4.75
> 1.85	23/117	1.88	0.85–4.39	1.79	0.79–4.28
P-trend			0.14		0.18
**By menopausal status:**					
**Premenopausal**					
< 1.26	18/19	1.0		1.0	
1.26–1.85	37/26	1.43	0.60–3.43	1.31	0.52.3.32
> 1.85	63/36	1.76	0.79–3.98	1.65	0.69–3.92
P-trend			0.10		0.19
**Postmenopausal**					
< 1.26	40/98	1.0		1.0	
1.26–1.85	64/95	1.66	1.01–2.74	1.62	0.97–2.73
> 1.85	76/81	2.32	1.42–3.83	2.06	1.23–3.50
P-trend			< 0.001		< 0.001

^a^Adjusted for age at diagnosis (cases) and age at interview (controls).

^b^Further adjusted for age at menarche, parity, menopausal status, and BMI.

### Potential modifiers

Table 4 shows the NLR-breast cancer association stratified by total cholesterol and urinary H_2_O_2_ levels. The NLR-breast cancer association was more pronounced for women with high total cholesterol levels (multivariable ORs (95% CI) = 2.79 (1.60–4.91), and 1.41 (0.66–3.04), P-trend < 0.001 and 0.09), for high and low total cholesterol levels, respectively), and low urinary H_2_O_2_ levels (multivariable ORs (95% CI) = 1.74 (0.59–5.43), and 3.30 (1.12–10.40), P-trend = 0.31 and 0.056, respectively), for high and low urinary H_2_O_2_ levels, respectively).

**Table 4:** Neutrophil to lymphocyte ratio (NLR) and risk of breast cancer according to total cholesterol and H_2_O_2_ levels.

	Total cholesterol < 193 mg/dL					Total cholesterol ≥ 193 mg/dL				
	Ca/Co	OR^a^	95% CI	OR^b^	95% CI	Ca/Co	OR^a^	95% CI	OR^b^	95% CI
**NLR**										
< 1.26	18/29	1.0		1.0		38/87	1.0		1.0	
1.26–1.85	22/32	1.10	0.49–2.47	0.91	0.39–2.10	75/85	1.99	1.20–3.34	1.96	1.14–3.38
> 1.85	48/48	1.59	0.78–3.32	1.41	0.66–3.04	85/62	3.01	1.80–5.10	2.79	1.60–4.91
P-trend			0.03		0.09			< 0.001		< 0.001

	H_2_O_2_ < 284 ng/mL					H_2_O_2_ ≥ 284 ng/mL				
	Ca/Co	OR^a^	95% CI	OR^b^	95% CI	Ca/Co	OR^a^	95% CI	OR^b^	95% CI
**NLR**										
< 1.26	9/17	1.0		1.0		9/22	1.0		1.0	
1.26–1.85	28/9	5.31	1.76–17.31	5.95	1.71–23.01	20/36	1.68	0.64–4.66	1.75	0.61–5.32
> 1.85	41/21	3.65	1.38–10.22	3.30	1.12–10.40	19/36	1.53	0.58–4.24	1.74	0.59–5.43
P-trend			0.016		0.056			0.31		0.31

^a^Adjusted for age at diagnosis (cases) and age at interview (controls).

^b^Further adjusted for age at menarche, parity, menopausal status, and BMI.

### Tumor grade, stage and histology

We also examined the NLR-breast cancer association by tumour grade, stage, and histology (Supplemental Table 1). Although we lacked precision, the NLR-breast cancer association seemed to be more pronounced among grades II and III than among grade I breast cancers (multivariable ORs (95% CI) for grades II and III versus grade I breast cancers were 1.99 (1.24–3.23), P-trend < 0.001, and 1.15 (0.53–2.56), P-trend = 0.07, respectively), and among stage 1 versus stages 2 and 3 breast cancers (multivariable ORs (95% CI) were 2.68 (1.41–5.32), P-trend < 0.001, and 1.81 (0.99–3.35), P-trend = 0.04 for stage 1 and stage 2 plus 3 breast cancer, respectively), although numbers were small. Although we lacked precision, the NLR-breast cancer association seemed to be more evident for ductal and lobular breast cancers (multivariable ORs (95% CI) = 2.07 (1.30–3.34), P-trend < 0.001, and 3.45 (1.02–15.76), P-trend = 0.002, respectively), than for mucinous/mixed breast cancer (multivariable OR (95% CI) = 0.46 (0.10–1.80), P-trend = 0.66).

We also examined the PLR-risk of breast cancer association in our study. We found an increased risk of breast cancer associated with the PLR highest category (multivariable OR (95% CI) = 1.68 (1.12–2.58), P-trend = 0.01). We also examined PLR by tumor grade, stage, and histology (Supplemental Table 1). Although we lacked precision, the PLR-breast cancer association seemed to be more pronounced among grades II and III (multivariable OR (95% CI) = 1.73 (1.10–2.74), P-trend = 0.008) than among grade I tumors (multivariable OR (95% CI) = 1.16 (0.59–2.31), P-trend = 0.89). No differences were detected by stage of disease (multivariable ORs for stage 1 versus stages 2 and 3 (95% CI) = 1.24 (0.72–2.19), P-trend = 0.36 and 1.36 (0.79–2.40), P-trend = 0.22, respectively). Similarly, although we lacked precision, the PLR-breast cancer association seemed to be slightly more apparent for ductal than for lobular or mucinous/mixed breast cancers (multivariable ORs (95% CI) = 1.59 (1.03–2.46), P-trend = 0.047, 2.03 (0.77–5.99), P-trend = 0.052, and 1.81 (0.46–8.82), P-trend = 0.63, for ductal, lobular and mucinous/mixed breast cancers, respectively).

## Discussion

In this study, we investigated the association between circulating WBC and the NLR, and the risk of breast cancer. To our knowledge, this is the first study examining the association between WBC and the NLR and breast cancer risk in non-Asian women, overall and by subtypes. Our findings indicate that breast cancer risk increased with increasing NE% and NLR among Luminal A, and HER2 negative breast cancers, and in both, pre- and postmenopausal women, although it was more pronounced among postmenopausal women. Similarly, higher L# and L% were associated with a decreased risk of breast cancer. No other WBC component was associated with risk of breast cancer.

There is abundant evidence for the adverse prognostic value of NLR on breast cancer. Multiple studies have shown that higher NLR was associated with adverse survival^4–9^, both disease-free survival and overall survival^11^ and more advanced or aggressive breast cancer^7,10,11^. However, only three prior studies, all conducted in Asian women, assessed the association between NLR and risk of breast cancer^7,17,18^. Two of the three studies compared breast cancer cases with patients with benign breast disease (BBD)^7,17^, and only one used healthy controls as comparison group^18^, as in our study. In one of the studies^17^, NLR was significantly higher in the breast cancer group compared with the BBD group, and patients with NLR > 1.67 were associated with an increased risk of breast cancer. This finding was consistent with those of the other two studies^7,18^, suggesting that NLR could be an independent risk factor for breast cancer. In one study, the best cut-point value for NLR was 2.96 and it was replicated, giving a sensitivity and a specificity of about 80% and 76%, respectively, in patients with breast cancer. In another study, the cut-point value for the NLR was 2.56.

Neutrophils are the most frequent (40% to 75%) type of white blood cells in mammals, and a crucial part of the innate immune system^22–24^. There is evidence for a detrimental role of neutrophils during tumour progression, but there is also solid evidence of an anticancer effect. Activated neutrophils can kill tumour cells in vitro^25^ and in vivo ^26^. Neutrophils only possess this antitumor effect after they have been activated. It was shown that a colon adenocarcinoma cell line transfected to express G-CSF (Granulocytic Colony Stimulating Factor) lost tumour activity after massive concentration of neutrophils at the tumour site^27^. In that study, purified neutrophils from SR/CR mice directly killed cancer cells in vitro and entirely conveyed resistance to recipient wild-type mice^27^. In addition, the cancer progressively disappeared after penetration of a big amount of neutrophils and a lesser amount of lymphocytes into the rest of the tumour tissues^28^. Neutrophils from tumour-bearing animals displayed enhanced reactive oxygen species (ROS) such as superoxide anion generation and phagocytosis, which led to reduced tumours and less metastatic foci in lungs^29,30^.

To do their anti-cancer effect, neutrophils have a remarkable ability to produce a high amount of ROS which is the phagocyte nicotinamide adenine dinucleotide phosphate (NADPH) oxidase^31,32^. In resting neutrophils cells, NADPH oxidase is inactive. Neutrophils migrate from blood to tissue and it is at the tissue level where they become activated through release of ROS such as H_2_O_2_, and then subsequently kill cancer cells. As neutrophils-induced ROS have not just been found extracellularly but also intracellularly^32,33^, accumulating that they participate as signalling molecules in cellular pathways such as apoptosis of tumour cells^34^.

This anticancer, pro-apoptotic, effect of activated neutrophils in tissue underlies our finding of an elevated risk of breast cancer with increasing levels of blood NE% and NLR. Higher neutrophil levels in blood, where they are inactive, may indicate a low corresponding concentration in tissues, where they are active. Inactive neutrophils are unable to release ROS or cause ROS-induced apoptosis of tumour cells. Neutrophils leave their patrol zone in the blood and go to the tissue where they commit suicide after becoming activated by releasing ROS and inducing apoptosis.

In the present study, we found NE%- and NLR-breast cancer associations to be more apparent when restricted to postmenopausal women. The mechanism underlying this finding is unknown. One possibility is reduced statistical power in premenopausal women, since, although we did find an increased risk in both, postmenopausal and premenopausal women, our study had smaller sample size and therefore limited precision for the latter (including 199 premenopausal and 454 postmenopausal women). Another possibility is that a different mechanistic pathway may exist between breast cancer patients and healthy controls by menopausal status, however, we found no evidence in the literature in support of this. A previous study conducted in Chinese women using BBD as controls examined the NLR-BC association by menopausal status and found that intermediate and high NLR levels significantly increased breast cancer risk compared to very low NLR levels in both, pre- and postmenopausal women^17^. In another study of ovarian cancer cases, total neutrophil counts and WBC were significantly increased in premenopausal women who were Jewish^35^, and premenopausal women who had a family history of breast or ovarian cancer also had higher NLR and lower lymphocyte counts^35^.

The rapid release of ROS such as superoxide radical and hydrogen peroxide, H_2_O_2_, by neutrophils is called oxidative burst and it may be responsible for their anticancer effect. We measured urinary hydrogen peroxide H_2_O_2_ in these patients and found that the detrimental effect of NLR on breast cancer was more pronounced among women with low levels of H_2_O_2_ in urine, as expected (multivariable ORs (95% CI) = 3.30 (1.12–10.40), P-trend = 0.056, and 1.74 (0.59–5.43), P-trend = 0.31 for low and high levels of H_2_O_2_, respectively).

### Potential modifiers

We also found that total cholesterol may modify the NLR-breast cancer relationships. Specifically, we found that the NLR effects on breast cancer risk were more pronounced in women with high levels of total cholesterol. One possible reason is that cholesterol inhibits NADPH oxidase, the crucial enzyme for ROS production and ROS-induced apoptosis of the cancer cell. NADPH oxidase catalyses a very intense production of superoxide ions O_2_^• −^ , which are transformed into another ROS such as H_2_O_2_. It has been found that one of the consequences of an increase of total cholesterol amount is an inhibition of the activity of NADPH oxidase in the presence of proinflammatory signals. This would imply a modification of the response to the signalling regulation^36^.

Breast cancer is much higher in women than men. Several prior studies have reported higher blood neutrophils rates in females than males^37,38^. In another study including young European women and men (median ages 25 years), neutrophil counts were observed to be higher in women^39^. Higher neutrophil counts in women were found in a study of Africans of 18–55 years of age^40^. In another study of healthy adults of several races, females had higher granulocyte counts but lower monocyte counts^37,41^.

Early age at menopause is an established protective factor for breast cancer, and, in healthy women, menopause decreases the NLR^37^. In a prior study, it has been shown that in healthy women aged around 50 years of age, there is an important drop of NE% and a significant rise of LY%, with a subsequent substantial fall in the NLR; however, in women older than 70 the NLR increased again^37^.

In healthy women, estradiol levels dramatically fall (by ~ 70%) during menopause, which commonly occurs around 50 years of age^42,43^. Estradiol has been shown to inhibit neutrophil apoptosis^44^, and decrease lymphocyte production in the bone marrow^45,46^. Therefore, in healthy women after menopause, the significant reduction of estradiol levels will likely result in higher neutrophil apoptotic rates and increased lymphocyte production, leading to reduced NE% and risen LY%, and reduced NLR compared to healthy premenopausal women. The neutrophil-apoptotic pathway could be one underlying mechanism behind the significant drop of NE% and substantial rise of LY% in women of around 50 years of age and that women of < 50 years of age have significantly higher NE% and lower LY% than women of 51–70 years of age from that study^37^. These findings, shown by this^37^ and other studies^47^, are also consistent with our own study results, as our mean neutrophil and NLR values in healthy women (controls) are higher in premenopausal than postmenopausal women (mean values for neutrophils and NLR in premenopausal controls were 3.84 ± 1.43 and 1.84 ± 0.75 respectively; the corresponding figures for postmenopausal controls are 3.29 ± 1.13 and 1.58 ± 0.68).

We also found an increased risk among both HER2-negative breast cancer, and, less pronounced, HER2-positive breast cancer. In a study, HER2 expression, among other factors (increased tumour size and PLR), was significantly associated with an elevated NLR^13,48^. HER2 over-expression occurs in approximately 15–30% of breast cancers^49^, and it is strongly associated with poor prognosis^50^. However, we found a more pronounced risk from NLR among HER2-negative breast cancer.

### NLR, ROS, and stem cell division

We will next describe the relationships between NLR, ROS and stem cell divisions. Peripheral blood neutrophils could be a marker of baseline apoptosis levels in tissues. Decreased peripheral blood neutrophils may indicate increased baseline apoptosis level in tissues, as a marker of the innate levels of ROS-induced apoptosis.

Among the mechanisms of tumour cell killing, activated neutrophils clearly have the potential of directly killing tumour cells, through ROS that can directly kill tumour cells. In addition to this direct anti-cancer effect, another cell killing mechanism relates to the immune system, also through the rapid release of ROS or the stimulation of T-cell response.

It has been shown that the number of endogenous mutations resulting from stem cell divisions correlates with cancer incidence^51^. Mesenchymal stem cells (MSCs) have been shown to suppress neutrophil activation through the inhibition of ROS release and subsequent apoptosis. Therefore, NE% and/or NLR could be a marker of the number of stem cell divisions. An increased number of stem cells divisions would imply a decreased activation of neutrophils and elevated numbers in blood^52^.

Human tumour macrophages express PD-L1^53^. PD-L1 functions as an immunomodulatory molecule, and it is broadly expressed in a variety of immune cells including neutrophils, T cells, B cells, dendritic cells, and monocytes. Increased NLR has been reported to be an independent poor prognostic indicator and its normalization following treatment has been found to predict progression-free survival in cancer patients undergoing treatment with PD-1/PD-L1 inhibitors^54^.

### Study limitations

Results of this study must be interpreted in light of its limitations. First, measurement of WBC can be difficult, and misclassification can occur. Our WBC levels were measured in standard clinical hospital laboratories, so measurement error is expected to be reduced. Also, any misreporting of blood WBC is unlikely to be influenced by tumour subtype. Residual confounding from alcohol intake, mammographic density, and female steroid hormones such as HRT can also influence the WBC-BC association. Also, for some breast cancer subtypes, such as HER2 overexpressing tumours, we lacked precision. Another limitation of our study was the lack of pre-diagnostic data from the cases to be able to conclude that NLR is positively related to breast cancer. Another limitation is lack of data on TILs in the tumors of our patients, which would strengthen the mechanistic pathway of apoptosis being one mechanism responsible for neutrophils anti-cancer effect. Another limitation is that we did not have enough follow-up on breast cancer survival or recurrence and thus could not explore the NLR-breast cancer effect on survival and/or recurrence, although this association has been confirmed by multiple studies. Conversely, strengths of our study are the direct measurements of WBC in our study population, and the available information on HER2, in addition to hormonal receptor status.

## Conclusion

We found elevated NLR to be associated with increased risk of breast cancer, principally Luminal A and HER2-negative breast cancer. To our knowledge, this is the first study examining the association between NLR and risk of breast cancer in non-Asian women, overall and by major breast cancer subtypes. The NLR-breast cancer associations appeared to be more pronounced among women with high levels of total cholesterol and low levels of H_2_O_2_, and among grades II and III than among grade I breast cancer. Neutrophils, which are inactive in blood, must migrate to tissue to become activated and liberate ROS to kill tumour cells, after which, they commit suicide and die. If there are increased neutrophils in blood it may indicate that they are not being sufficiently activated and therefore not releasing ROS, implying low ROS levels in tissues. Our finding that increased NE% and NLR in blood is associated with increased risk of breast cancer may indicate that neutrophils are not being sufficiently activated in breast tissue to induce neutrophil-induced apoptosis. Increased peripheral blood neutrophils could be a marker of decreased baseline or innate apoptosis levels in tissues.

## Materials and methods

### Study population

The Breast Oncology Galician Network (BREOGAN) includes a population-based case–control study conducted in the cities of Santiago de Compostela and Vigo, Spain, within a geographically defined health region that covers approximately one million inhabitants. Data collection methods have been previously described^19–21,55–57^. Cases comprised 1,766 women with invasive breast cancer diagnosed and treated between 1997 and 2014 at the Clinical University Hospitals of Vigo (CHUVI) and Santiago (CHUS), as described in previous studies. Controls were 1,205 women free of cancer, except non-melanoma skin cancer, living in the same population health area as cases. Response rates were 98% and 99% for cases and controls, respectively. Ethics approval for this study was obtained from the CEIC, Comité Ético de Investigación Clínica de Galicia (Galician Ethics and Research Committee), responsible for the oversight of both university hospitals, CHUVI and CHUS, and family, primary clinics from where all participants were recruited. All participants provided written informed consent. The study was conducted in accordance to the Helsinki Principles of 1975, as revised in 1983.

### Data collection

#### Risk factor data

Similar to previous studies^19–21,56^, risk factor information was collected through a risk factor questionnaire adapted from the *Ella* Binational Breast Cancer Study^56,58,59^ to meet the needs of the population in Spain. Clinical and histopathological information was abstracted from computerized medical records by trained physicians. The following variables were recorded: level of education [uneducated (less than primary education), primary education, secondary education, vocational training, 3-years degree (certificate, middle engineering), 5-years degree (graduate school, bachelor´s degree, superior engineering), and PhD (doctorate)], lifetime breastfeeding [categorized as no breastfeeding, < lifetime breastfeeding duration (12 months), ≥ lifetime breastfeeding duration (12 months)], age at menarche, age at first full-term pregnancy, parous (categorized as never vs. ever pregnant), number of pregnancies (parity, categorized as none, 1–2, ≥ 3), age at menopause (≤ 50, > 50), menopausal status at diagnosis (categorized as pre and postmenopausal), oral contraceptive use (never, ever), hormone replacement therapy (HRT, never, ever), smoking status (never smoker, ex-smoker, current smoker), family history (categorized as none vs. one or more first and/or second degree relatives with breast and/or ovarian cancer). Alcohol consumption was evaluated by the number of alcoholic drinks consumed regularly per week in last year before reference date. The alcohol categories correspond to the CDC definition (https://www.cdc.gov/alcohol/faqs.htm#heavyDrinking) of light/moderate and heavy drinking among women.

Data on circulating WBC including neutrophils, monocytes, and lymphocytes before date of diagnosis for cases and date of interview for controls were abstracted from Ianus, the Galician universal computerized medical history. WBC count was done through automated machine counting of cells. Briefly, withdrawal of peripheral blood by venipuncture was performed during fasting. Total WBC and differential counts were performed on the peripheral blood samples by using the ADVIA 2120 Hematology analyzer (Siemens Healthcare Diagnostics, Tarrytown, NY). We calculated neutrophil to lymphocyte ratio (NLR) by dividing the number of neutrophils by number of lymphocytes from peripheral blood sample.

#### Clinic-pathological data

Similar to our previous studies^19–21,56^, histopathological information was abstracted from computerized medical records by trained physicians. Immunohistochemistry (IHC) analyses on paraffin-embedded material have been previously performed following standard procedures in Galician hospitals to determine the status of ER and PR. In every tumour, 4-μm histological sections were cut and stained with haematoxylin and eosin for histopathological examination according to the criteria of the World Health Organization^60^. Histological grading was evaluated using the Nottingham modification of the Bloom-Richardson system^61^. Similar to previous studies^19–21,56^, immunohistochemistry (IHC) analysis on paraffin-embedded material was performed using a universal second antibody kit that used a peroxidase-conjugated labelled dextran polymer (EnVision®, Peroxidase/DAB,Dako, Glostrup, Denmark), with antibodies for ER (clone 6F11, dilution 1:50, water bath,Novocastra, Newcastle-upon- Tyne, UK), PR (clone PgR 636, dilution 1:50, water bath,Dako, Glostrup, Denmark). Negative and positive controls were concurrently run for all antibodies with satisfactory results. Cells were considered immunopositive when diffuse or dot-like nuclear staining was observed regardless of the intensity of the staining,only nuclear immunoreactivity was considered specific. The number of positive cells was counted by two different observers independently. Whenever necessary, a consensus was reached using a double-headed microscope. ER and PR were considered positive when the percent of immunostained nuclei was ≥ 10%.

Similar to our previous studies^19–21,56^, IHC analyses were performed to determine HER2 status (Dako). No immunostaining (0) or weak membrane immunostaining (1 +) was considered low HER2 expression (HER2). Strong membrane immunostaining (3 +) was considered HER2 overexpression (HER2 +). Moderate membrane staining (2 +samples were further analysed using fluorescence in situ hybridization techniques; they were considered to be HER2 + if the ratio of cerb-B2/centromere 17 copy number was > 2.0.

Similar to previous studies^19–21,56^, ER, PR and HER2 status (categorized as positive and negative), grade (categorized as I – well differentiated –, II – moderately differentiated– and III – poorly differentiated or undifferentiated), histology type (categorized as invasive ductal carcinoma, invasive lobular carcinoma and other), and tumour size (cm). As previously described in previous studies^21^, of the 1766 women who participated in the study, 100 had unknown ER status, 114 had unknown PR status, and 340 had unknown HER2 status. In the same manner, one hundred and eighty-four women had unknown grade, 14 had unknown histological type and 144 had unknown tumour size^21^. Sixty-two women had unknown age at menarche, and 48, out of 1,443 parous women, had unknown lifetime breastfeeding^21^. Urinary H_2_O_2_ was measured by ELISA test using K034-F1 Hydrogen Peroxide Fluorescent Detection Kit, de Arbor, 339,00 Arbor Assays DetectX® Hydrogen Peroxide Fluorescent Detection Assay, with all 4 °C stable reagents.

### Statistical analyses

The association of breast cancer with circulating WBC and NLR was measured by odds ratios (ORs) and corresponding 95% confidence intervals (CIs) using unconditional polytomous logistic regression. Analyses were initially adjusted for the following established risk or protective factors for breast cancer: reference age (age at diagnosis for cases and age at interview for controls), age at menarche, parity, breastfeeding, menopausal status, weight, height, oral contraceptive use, hormone replacement therapy and family history of first-degree relatives with breast and/or ovarian cancer. Results were virtually unchanged after adjustment for all these variables or only age, age at menarche, parity, menopausal status, and BMI, therefore we present results adjusted for the latter. Outcome (dependent) variables were breast cancer subtypes defined by ER, PR, and HER2 status (we defined four tumour subtypes (ER + /HER2- or PR + /HER2- [Luminal A], ER + /HER2 + or PR + /HER2 + [Luminal B], ER-/PR-/HER2 + [HER2 overexpressing or HER2 +], and ER-/PR-/HER2-[TNBC])), compared to controls (comparison group), and explanatory variables were circulating WBC (neutrophils, monocytes, lymphocytes, and NLR). Cutoff points for subgroup analysis, i.e., NLR, PLR, total cholesterol and urinary H_2_O_2_, were calculated based on distribution among controls. Briefly, cut points for NLR, based on tertile distribution among control group, were comparable to cut points based on previous studies on the effect of the NLR on breast cancer survival and/or prognosis^4–11,13^. Similarly, cut points for PLR were based on tertile distribution among control group, which were comparable to cut points based on previous studies on the effect of the PLR on breast cancer^62^. Cut points for serum total cholesterol levels were based on tertile distribution among the control group (< 193 mg/dL, ≥ 193 mg/dL), which are equivalent to standard levels of normal and borderline/high total cholesterol levels (< 200 mg/dL, > 200 mg/dL). Cut points for urinary H_2_O_2_ levels were also based on tertile distribution among the control group (< 284 ng/mL, ≥ 284 ng/mL). All statistical analyses were performed using the R statistical software version 3.3.3. All reported test significance levels (P values) were two-sided.

## Supplementary information

Supplementary information.
