# Author Correction: Neutrophil to lymphocyte ratio and breast cancer risk: analysis by subtype and potential interactions

**DOI:** 10.1038/s41598-020-75458-y

**Published:** 2020-11-20

**Authors:** Manuela Gago‑Dominguez, Marcos Matabuena, Carmen M. Redondo, Sandip Pravin Patel, Angel Carracedo, Sara Miranda Ponte, María Elena Martínez, J. Esteban Castelao

**Affiliations:** 1 Galician Public Foundation of Genomic Medicine (FPGMX), Genomic Medicine Group, International Cancer Genetics and Epidemiology Group, Health Research Institute of Santiago (IDIS), Santiago de Compostela, Spain; 2 Moores Cancer Center, University of California San Diego, La Jolla, CA USA; 3 Centro de Investigación en Tecnoloxías da Información (CITIUS), Universidade de Santiago de Compostela, Santiago de Compostela, Spain; 4 Oncology and Genetics Unit, Instituto de Investigación Sanitaria Galicia Sur, Vigo, Spain; 5 Forensic Department, Universidade de Santiago de Compostela, Santiago de Compostela, Spain; 6 Department of Family Medicine and Public Health, University of California San Diego, La Jolla, CA USA

Correction to: *Scientific Reports* 10.1038/s41598-020-70077-z, published online 06 August 2020

In the original version of this Article, J. Esteban Castelao and Sara Miranda Ponte were incorrectly affiliated with ‘Centro de Investigación en Tecnoloxías da Información (CITIUS), Universidade de Santiago de Compostela, Santiago de Compostela, Spain’. The correct affiliation is listed below.

Oncology and Genetics Unit, Instituto de Investigación Sanitaria Galicia Sur, Vigo, Spain

This error has now been corrected in the PDF and HTML versions of the Article, and in the accompanying Supplementary Information.

