# Supplementary material for: Neutrophil to lymphocyte ratio and breast cancer risk: analysis by subtype and potential interactions
Source: Sci Rep. 2020 Aug 6;10:13203. doi: 10.1038/s41598-020-70077-z (PMC7413522; doi:10.1038/s41598-020-70077-z)
Supplement: Supplementary file 1 — Supplementary information. [file 41598_2020_70077_MOESM1_ESM.docx]

**Neutrophil to Lymphocyte Ratio and Breast Cancer Risk: Analysis by Subtype**

**and Potential Interactions**

**Manuela Gago-Dominguez^1,2*^**

Corresponding author

E-mail: [manuela.gago.dominguez@sergas.es](mailto:manuela.gago.dominguez@sergas.es)

**Marcos Matabuena**^3^

E-mail: marcos.matabuena@usc.es

**Carmen M. Redondo**^4^

E-mail: [carmen.redondo.marey](mailto:carmen.redondo.marey@sergas.es)@[sergas.es](mailto:carmen.redondo.marey@sergas.es)

**Sandip Pravin Patel**^2^

E-mail: patel@ucsd.edu

**Angel Carracedo^1, 5^**

E-mail: angel.carracedo@usc.es

**Sara Miranda Ponte**^3^

E-mail: [sara.miranda.ponte](mailto:sara.miranda.ponte@sergas.es)@[sergas.es](mailto:sara.miranda.ponte@sergas.es)

**María Elena Martínez**^2,6^

E-mail: e8martinez@ucsd.edu

**J. Esteban Castelao**^3^

E-mail: [jose.esteban.castelao.fernandez@sergas.es](mailto:jose.esteban.castelao.fernandez@sergas.es)

^1^ Galician Public Foundation of Genomic Medicine (FPGMX), Genomic Medicine Group, Health Research Institute of Santiago (IDIS), Santiago de Compostela, Spain. ^2^ Moores Cancer Center, University of California, San Diego, La Jolla, California, USA. ^3^ Centro de Investigación en Tecnoloxías da Informacion (CITIUS), Universidade de Santiago de Compostela, Santiago de Compostela, Spain. ^4^ Oncology and Genetics Unit, CHUVI Hospital. Fundación Biomédica Galicia-Sur, SERGAS, Vigo, Spain. ^5^ Forensic Department Universidade de Santiago de Compostela, Santiago de Compostela, Spain. ^6^ Department of Family Medicine and Public Health University of California, San Diego, La Jolla, California, USA.

**Corresponding author: Manuela Gago-Dominguez,** Health Research Institute of Santiago IDIS, Genomic Medicine Group, Galician Foundation of Genomic Medicine, Complejo Hospitalario Universitario de Santiago, SERGAS, Santiago de Compostela, Spain.

| **Supplemental Table 1. NLR and PLR and Risk of Breast Cancer by Grade, Stage, and Histology** | | | | | | | | | |
| --- | --- | --- | --- | --- | --- | --- | --- | --- | --- |
|  |  |  |  |  |  |  |  |  |  |
|  | **Cases N** | **Controls N** | **OR*** | **95% CI** | **Cases N** | **Controls N** | **OR**** | **95% CI** |  |
|  |  |  |  |  |  |  |  |  |  |
| **NLR** |  |  |  |  |  |  |  |  |  |
|  |  |  |  |  |  |  |  |  |  |
|  |  |  |  |  |  |  |  |  |  |
| **Grade 1** |  |  |  |  |  |  |  |  |  |
| < 1.26 | 13 | 117 | 1.00 |  | 13 | 106 | 1.00 |  |  |
| 1.26-1.85 | 24 | 121 | 1.70 | 0.83-3.60 | 23 | 111 | 1.38 | 0.65-3.02 |  |
| > 1.85 | 23 | 117 | 1.58 | 0.77-3.39 | 22 | 105 | 1.15 | 0.53-2.56 |  |
| P-trend |  |  |  | 0.01 |  |  |  | 0.07 |  |
|  |  |  |  |  |  |  |  |  |  |
| **Grades 2 and 3** |  |  |  |  |  |  |  |  |  |
| < 1.26 | 41 | 117 | 1.00 |  | 39 | 106 | 1.00 |  |  |
| 1.26-1.85 | 67 | 121 | 1.54 | 0.96-2.48 | 67 | 111 | 1.47 | 0.91-2.42 |  |
| > 1.85 | 93 | 117 | 2.11 | 1.34-3.34 | 90 | 105 | 1.99 | 1.24-3.23 |  |
| P-trend |  |  |  | < 0.001 |  |  |  | < 0.001 |  |
|  |  |  |  |  |  |  |  |  |  |
|  |  |  |  |  |  |  |  |  |  |
| **Stage 1** |  |  |  |  |  |  |  |  |  |
| < 1.26 | 15 | 117 | 1.00 |  | 15 | 106 | 1.00 |  |  |
| 1.26-1.85 | 25 | 121 | 1.57 | 0.79-3.19 | 24 | 111 | 1.39 | 0.69-2.88 |  |
| > 1.85 | 45 | 117 | 2.83 | 1.52-5.53 | 44 | 105 | 2.68 | 1.41-5.32 |  |
| P-trend |  |  |  | < 0.001 |  |  |  | < 0.001 |  |
|  |  |  |  |  |  |  |  |  |  |
| **Stage 2 and 3** |  |  |  |  |  |  |  |  |  |
| < 1.26 | 21 | 117 | 1.00 |  | 21 | 106 | 1.00 |  |  |
| 1.26-1.85 | 51 | 121 | 2.29 | 1.30-4.13 | 50 | 111 | 1.95 | 1.09-3.59 |  |
| > 1.85 | 54 | 117 | 2.38 | 1.36-4.28 | 51 | 105 | 1.81 | 0.99-3.35 |  |
| P-trend |  |  |  | 0.002 |  |  |  | 0.04 |  |
|  |  |  |  |  |  |  |  |  |  |
|  |  |  |  |  |  |  |  |  |  |
| **Histology Ductal** |  |  |  |  |  |  |  |  |  |
| < 1.26 | 41 | 117 | 1.00 |  | 41 | 106 | 1.00 |  |  |
| 1.26-1.85 | 77 | 121 | 1.73 | 1.09-2.78 | 77 | 111 | 1.53 | 0.95-2.49 |  |
| > 1.85 | 113 | 117 | 2.47 | 1.58-3.90 | 106 | 105 | 2.07 | 1.30-3.34 |  |
| P-trend |  |  |  | < 0.001 |  |  |  | < 0.001 |  |
|  |  |  |  |  |  |  |  |  |  |
| **Histology Lobular** |  |  |  |  |  |  |  |  |  |
| < 1.26 | 4 | 117 | 1.00 |  | 3 | 106 | 1.00 |  |  |
| 1.26-1.85 | 11 | 121 | 2.62 | 0.87-9.66 | 9 | 111 | 2.81 | 0.80-13.07 |  |
| > 1.85 | 12 | 117 | 2.88 | 0.97-10.58 | 12 | 105 | 3.45 | 1.02-15.76 |  |
| P-trend |  |  |  | 0.003 |  |  |  | 0.002 |  |
|  |  |  |  |  |  |  |  |  |  |
| **Histology Mucinous and Mixed** |  |  |  |  |  |  |  |  |  |
| < 1.26 | 6 | 117 | 1.00 |  | 6 | 106 | 1.00 |  |  |
| 1.26-1.85 | 7 | 121 | 1.14 | 0.39-3.65 | 7 | 111 | 1.01 | 0.31-3.34 |  |
| > 1.85 | 4 | 117 | 0.68 | 0.17-2.45 | 4 | 105 | 0.46 | 0.10-1.80 |  |
| P-trend |  |  |  | 0.89 |  |  |  | 0.66 |  |
|  |  |  |  |  |  |  |  |  |  |
| **Histology Other** |  |  |  |  |  |  |  |  |  |
| < 1.26 | 4 | 117 | 1.00 |  | 3 | 106 | 1.00 |  |  |
| 1.26 - 1.85 | 4 | 121 | 0.96 | 0.22-4.17 | 4 | 111 | 0.98 | 0.20-5.24 |  |
| > 1.85 | 4 | 117 | 1.00 | 0.23-4.33 | 4 | 105 | 0.70 | 0.11-4.23 |  |
| P-trend |  |  |  | 0.91 |  |  |  | 0.58 |  |
|  |  |  |  |  |  |  |  |  |  |
|  |  |  |  |  |  |  |  |  |  |
| **PLR** |  |  |  |  |  |  |  |  |  |
|  |  |  |  |  |  |  |  |  |  |
|  |  |  |  |  |  |  |  |  |  |
| **Grade 1** |  |  |  |  |  |  |  |  |  |
| < 95.7 | 19 | 117 | 1.00 |  | 19 | 107 | 1.00 |  |  |
| 95.7 - 126 | 12 | 112 | 0.66 | 0.29-1.41 | 12 | 101 | 0.64 | 0.28-1.41 |  |
| > 126 | 29 | 128 | 1.36 | 0.72-2.60 | 27 | 116 | 1.16 | 0.59-2.31 |  |
| P-trend |  |  |  | 0.56 |  |  |  | 0.89 |  |
|  |  |  |  |  |  |  |  |  |  |
| **Grades 2 and 3** |  |  |  |  |  |  |  |  |  |
| < 95.7 | 49 | 117 | 1.00 |  | 46 | 107 | 1.00 |  |  |
| 95.7 - 126 | 59 | 112 | 1.31 | 0.82-2.09 | 59 | 101 | 1.40 | 0.86-2.29 |  |
| > 126 | 94 | 128 | 1.71 | 1.11-2.65 | 92 | 116 | 1.73 | 1.10-2.74 |  |
| P-trend |  |  |  | 0.005 |  |  |  | 0.008 |  |
|  |  |  |  |  |  |  |  |  |  |
|  |  |  |  |  |  |  |  |  |  |
| **Stage 1** | 28 | 117 | 1.00 |  | 27 | 107 | 1.00 |  |  |
| < 95.7 |  |  |  |  |  |  |  |  |  |
| 95.7 - 126 | 17 | 112 | 0.64 | 0.33-1.23 | 16 | 101 | 0.59 | 0.29-1.15 |  |
| > 126 | 40 | 128 | 1.30 | 0.75-2.26 | 39 | 116 | 1.24 | 0.72-2.19 |  |
| P-trend |  |  |  | 0.30 |  |  |  | 0.36 |  |
|  |  |  |  |  |  |  |  |  |  |
| **Stage 2 and 3** |  |  |  |  |  |  |  |  |  |
| < 95.7 | 29 | 117 | 1.00 |  | 28 | 107 | 1.00 |  |  |
| 95.7 - 126 | 47 | 112 | 1.71 | 1.00-2.95 | 47 | 101 | 1.66 | 0.95-2.94 |  |
| > 126 | 50 | 128 | 1.53 | 0.91-2.62 | 47 | 116 | 1.36 | 0.79-2.40 |  |
| P-trend |  |  |  | 0.06 |  |  |  | 0.22 |  |
|  |  |  |  |  |  |  |  |  |  |
|  |  |  |  |  |  |  |  |  |  |
| **Histology Ductal** | 58 | 117 | 1.00 |  | 57 | 107 | 1.00 |  |  |
| < 95.7 |  |  |  |  |  |  |  |  |  |
| 95.7 - 126 | 62 | 112 | 1.16 | 0.74-1.83 | 61 | 101 | 1.16 | 0.72-1.87 |  |
| > 126 | 111 | 128 | 1.69 | 1.12-2.59 | 106 | 116 | 1.59 | 1.03-2.46 |  |
| P-trend |  |  |  | 0.01 |  |  |  | 0.047 |  |
|  |  |  |  |  |  |  |  |  |  |
| **Histology Lobular** |  |  |  |  |  |  |  |  |  |
| < 95.7 | 7 | 117 | 1.00 |  | 6 | 107 | 1.00 |  |  |
| 95.7 - 126 | 5 | 112 | 0.74 | 0.21-2.40 | 4 | 101 | 0.77 | 0.19-2.82 |  |
| > 126 | 15 | 128 | 1.93 | 0.78-5.20 | 14 | 116 | 2.03 | 0.77-5.99 |  |
| P-trend |  |  |  | 0.02 |  |  |  | 0.052 |  |
|  |  |  |  |  |  |  |  |  |  |
| **Histology Mucinous and Mixed** |  |  |  |  |  |  |  |  |  |
| < 95.7 | 3 | 117 | 1.00 |  | 3 | 107 | 1.00 |  |  |
| 95.7 - 126 | 7 | 112 | 2.43 | 0.66-11.51 | 7 | 101 | 2.56 | 0.68-12.36 |  |
| > 126 | 7 | 128 | 2.14 | 0.58-10.13 | 7 | 116 | 1.81 | 0.46-8.82 |  |
| P-trend |  |  |  | 0.96 |  |  |  | 0.63 |  |
|  |  |  |  |  |  |  |  |  |  |
| **Histology Other** | 2 | 117 | 1.00 |  | 1 | 107 | 1.00 |  |  |
| < 95.7 |  |  |  |  |  |  |  |  |  |
| 95.7 - 126 | 7 | 112 | 3.66 | 0.86-24.95 | 7 | 101 | 6.31 | 1.06-120.29 |  |
| > 126 | 4 | 128 | 1.81 | 0.35-13.25 | 4 | 116 | 3.02 | 0.42-60.50 |  |
| P-trend |  |  |  | 0.13 |  |  |  | 0.10 |  |
|  |  |  |  |  |  |  |  |  |  |
| * Adjusted for age at diagnosis (cases) and age at interview (controls). | | | | | | | | | |
| **Further adjusted for age at menarche, parity, menopausal status, and BMI. | | | | | | | | | |
|  | | | | | | | | | |
